# The complete chloroplast genome of *Philodendron hederaceum* (Jacq.) Schott 1829 (Alismatales: Araceae)

**DOI:** 10.1080/23802359.2024.2311748

**Published:** 2024-02-09

**Authors:** Gyoungju Nah, Ji Ran Jeong, Jae Hwan Lee, Soon Yil Soh, Sang Yong Nam

**Affiliations:** a Genome Analysis Center at National Instrumentation Center for Environmental Management, Seoul National University, Seoul, South Korea; b Department of Environmental Horticulture, Sahmyook University, Seoul, South Korea; c Natural Science Research Institute, Sahmyook University, Seoul, South Korea

**Keywords:** Araceae, complete chloroplast genome, next-generation sequencing, Oxford Nanopore sequencing, *Philodendron hederaceum*

## Abstract

*Philodendron hederaceum* (Jacq.) Schott 1829, a species of the Araceae family, is a foliage plant of ornamental value. The complete chloroplast genome sequence of *Philodendron hederaceum* was obtained by the *de novo* assembly of NovaSeq 6000 (Illumina Co., San Diego, CA) paired-end short reads and Oxford Nanopore long reads. The complete chloroplast genome of *P. hederaceum* was 168,609 bp in length, with a large single-copy (LSC) region of 94,393 bp, a small single-copy (SSC) region of 25,774 bp, and a pair of identical inverted repeat regions (IRs) of 24,221 bp. The genome contained a total of 129 genes, including 85 protein-coding genes, 36 transfer RNA (tRNA) genes, and eight ribosomal RNA (rRNA) genes. The phylogenetic analysis of *P. hederaceum* with 19 related species and two outgroup species revealed the closest taxonomical relationship with *Philodendron lanceolatum* in the Araceae family.

## Introduction

*Philodendron* is the second-largest genus belonging to the Araceae, the tropical monocot family characterized by diverse morphology and a wide geographic distribution in the Neotropics (Loss-Oliveira et al. 2016; Goncalves and Mayo 2000).

The *Philodendron* genus comprises 482 species (Boyce and Croat 2018). Its geographic distribution, spanning from Northern Mexico to Southern Uruguay (Mayo et al. 1997), contributes to the formation of rainforests in the Amazon and South America. The species belonging to the *Philodendron* genus are mainly distributed in Brazil, where 168 species were reported (Coelho et al. 2016). While the inflorescence morphology of *Philodendron* is generally conserved, the leaf morphology and habitats of *Philodendron* exhibit a wide range of diversity (Coelho 2000; Coelho et al. 2016). *Philodendron* species are classified into three subgenera based on floral and vegetative morphology (Mayo 1991; Croat 1997): the subgenus *Meconostigma* (Schott) Engl. (Gonçalves and Salviani 2002), the subgenus *Pteromischum* (Schott) Mayo (Coelho 2000), and the subgenus *Philodendron* (Coelho 2000).

*P. hederaceum* (Jacq.) Schott 1829, also known as the heart-leaf philodendron, is native to Central America and the Caribbean. It is characterized by bright glossy heart-shaped dark green leaves and is used as an ornamental plant. *P. hederaceum* is an evergreen climber that reaches a height of 3–6 m and occasionally produces yellow-green and white flowers (Goncalves and Mayo 2000), adding to its ornamental appeal. This study presents the complete chloroplast genome of *Philodendron hederaceum*, aiming to explore further phylogenetic relationships within the *Philodendron* genus.

## Materials and methods

**Figure 1.**
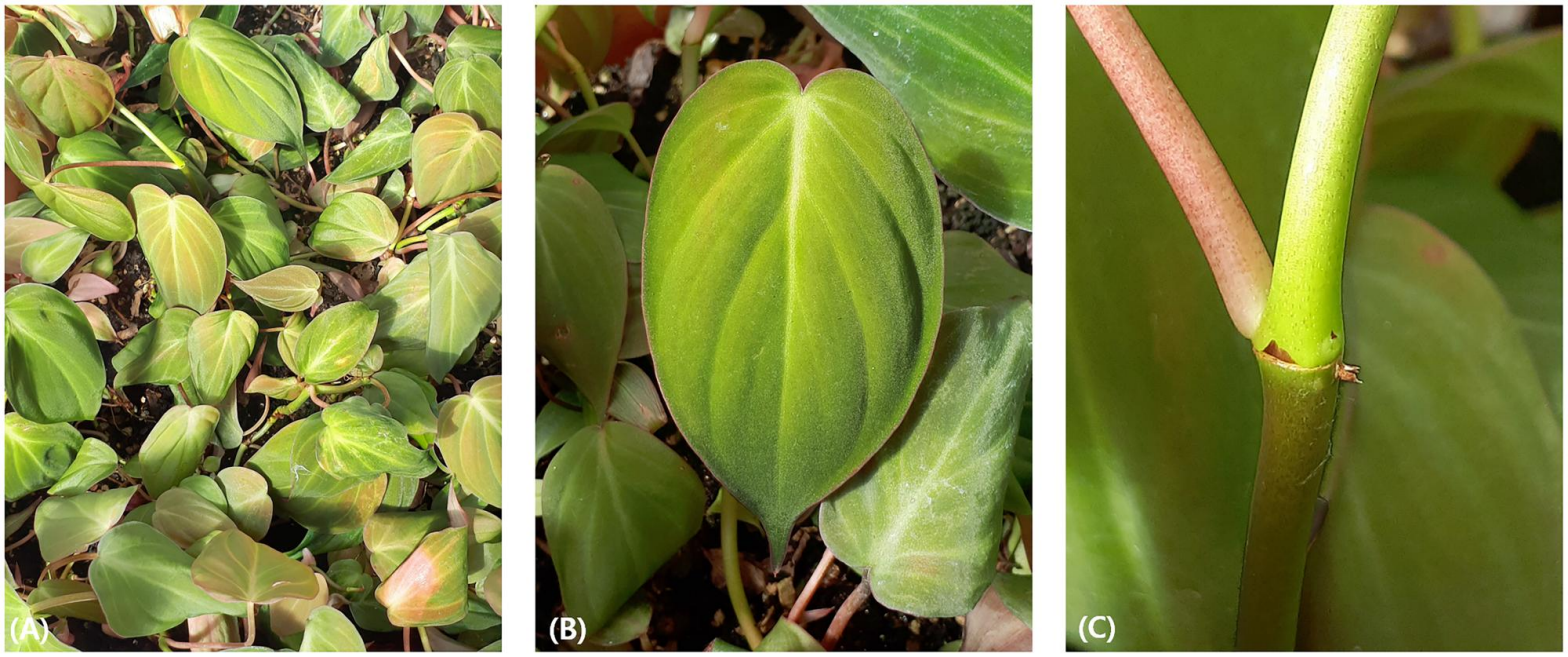
Photograph of *Philodendron hederaceum* (Jacq.) Schott 1829 (these photographs were taken by Jae Hwan Lee). The foliage of *P. hederaceum* exhibits heart-shape and glossy texture on cascading stems. Upper surface of leaves has medium to dark green color, while lower surface has medium green color. The primary lateral veins have 2–6 per side, and emerge from the midvein at an angle of 35° to 55°. (A) Plants of *P. hederaceum*, (B) the leaf of *P. hederaceum*, and (C) the stem of *P. hederaceum.*

The *P. hederaceum* plants were initially collected in Ciudad de Panamá, Panama (9°01′40″N 79°33′50″W), following the local regulations and with the necessary permissions from local authorities. Subsequently, the collected *P. hederaceum* plants were propagated and are currently maintained in the greenhouse at the Natural Science Research Institute, Sahmyook University, Seoul, South Korea (Figure 1). The voucher specimen with the collection number SYFS101 has been deposited at the Natural Science Research Institute, Sahmyook University, Seoul, South Korea (https://www.syu.ac.kr/natural-science-institute/, contact person: Jae Hwan Lee, dlwoghks1236@naver.com). This institute is affiliated with the National Agrobiodiversity Center at Rural Development Administration, Jeonju, South Korea, and serves as the institute for agricultural genetic resources.

The genomic DNA was extracted from the leaf tissues of *P. hederaceum* using a modified CTAB (cetrimonium bromide)-based protocol. Subsequently, DNA quantitation and integrity were validated using Nanodrop (Thermo Scientific Inc., Waltham, MA) and agarose gel electrophoresis. The construction of the Illumina paired-end (PE) genomic library was performed using the NEXTflex^®^ Rapid DNA sequencing kit (Bioo Scientific, Austin, TX), following the manufacturer’s protocol. The chloroplast genome of *P. hederaceum* was sequenced using NovaSeq6000 (Illumina Inc., San Diego, CA), and subsequently, low-quality and adaptor sequences were removed with Trimmomatic (Bolger et al. 2014). To circularize the chloroplast genome, additional long-read sequences were generated using Oxford Nanopore Promethion24 (Oxford Nanopore Technologies Inc., Oxford, UK), following the manufacturer’s recommendations for constructing the long-read sequencing library.

To complete the chloroplast genome assembly, we constructed a hybrid assembly using both Illumina and Nanopore sequences with an Organelle PBA assembler (Soorni et al. 2017). Subsequently, error correction of the final contig was carried out using Illumina reads with NextPolish (ver. 1.3.0, https://github.com/Nextomics/NextPolish). The gene prediction for the assembled chloroplast genome was executed using GeSeq (Tillich et al. 2017) with subsequent manual correction. Following annotation, the entire chloroplast genome sequence of *P. hederaceum* was submitted to GenBank under the accession number OM937109.

To investigate the phylogenetic position of *P. hederaceum*, we obtained the complete chloroplast genome sequences of 14 related species in Araceae from GenBank, along with two outgroup species (*Sagittaria lichuanensis* and *Thalassia hemprichii*). The sequences were aligned using ClustalW (ver. 2.1) (Larkin et al. 2007). Subsequently, a phylogenetic tree was constructed through the maximum-likelihood (ML) method with 1000 bootstraps, utilizing MEGA 10.2.5 (Kumar et al. 2016).

## Results

**Figure 2.**
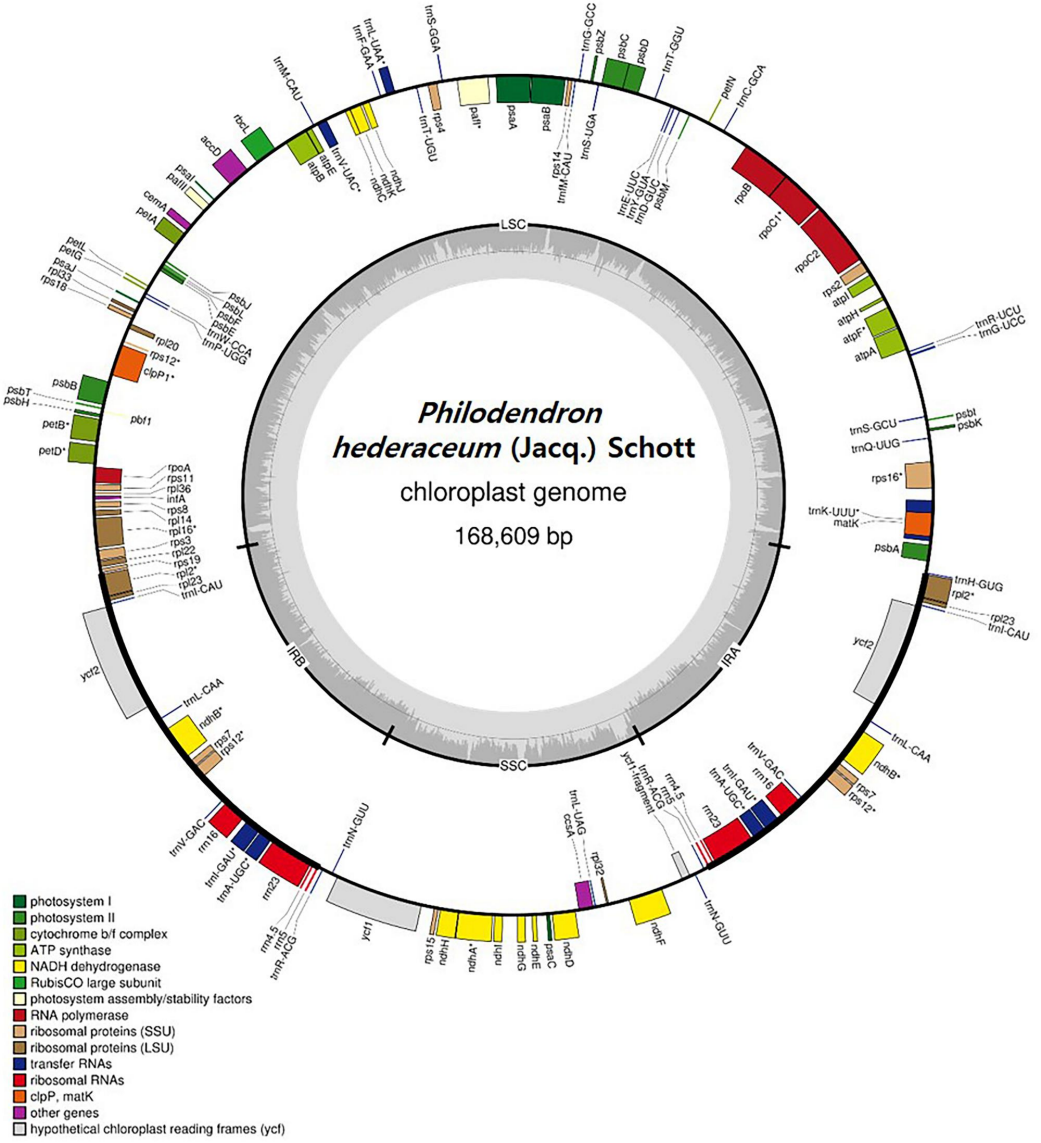
Chloroplast genome map of *Philodendron hederaceum*. Genes inside the circle are transcribed in a counterclockwise direction, and genes outside the circle are transcribed in a clockwise direction. The color of each gene represents its function. LSC, SSC, IRa, and IRb regions are indicated in this map.

The complete chloroplast genome (OM937109) of *P. hederaceum* was assembled using Nanopore sequencing reads with an average depth of 431.49× (minimum 30× and maximum 1039× depth) (Figure S1), followed by error correction with Illumina reads. The length of the circularized chloroplast genome was measured to be 168,609 bp with a G + C content of 35.17% (Figure 2). The genome comprises a large single-copy (LSC) region of 94,393 bp, a small single-copy (SSC) region of 25,774 bp, and a pair of inverted repeat regions (IRa and IRb) of 24,221 bp. Additionally, the genome harbors a total of 129 genes, encompassing 85 protein-coding genes, 36 tRNA genes, and eight rRNA genes (Figure 2). These 129 predicted genes were categorized into 21 groups, as listed in Table 1. Among them, eight genes (*acc*D, *ccs*A, *cem*A, *clp*P1, *inf*A, *mat*K, *pbf*1, *rbc*L1) were identified as single-copy genes, while the remaining genes exist as duplicated forms (Table 1). Additionally, 13 cis-splicing genes (*atp*F, *clp*PI, *paf*I, *pe*tB, *pet*D, *rpl*2x2, *rpl*16*, rpo*C1, *rps*16, *ndh*A, *ndh*Bx2) and one trans-splicing gene (*rps*12) were identified in this analysis (Figure S2).

**Table 1.** Gene prediction of *Philodendron hederaceum* chloroplast genome.

Category	No.	Gene
Acetyl-CoA carboxylase	1	*acc*D
Subunits of ATP synthase	6	*atp*A, *atp*B, *atp*E, *atp*F, *atp*H, *atp*I
Cytochrome c biogenesis	1	*ccs*A
Envelope membrane protein	1	*cem*A
Protease	1	*clp*P1
Translation initiation factor	1	*inf*A
Maturase	1	*mat*K
Subunits of NADH dehydrogenase	12	*ndh*A, *ndh*B (x2), *ndh*C, *ndh*D, *ndh*E, *ndh*F, *ndh*G, *ndh*H, *ndh*I, *ndh*J, *ndh*K
Photosystem I assembly factor	2	*paf*I, *paf*II
Photosystem II assembly factor	1	*pbf*1
Subunits of cytochrome b/f complex	6	*pet*A, *pet*B, *pet*D, *pet*G, *pet*L, *pet*N
Subunits of photosystem I	5	*psa*A, *psa*B, *psa*C, *psaI*, *psa*J
Subunits of photosystem II	14	*psb*A, *psb*B, *psb*C, *psb*D, *psb*E, *psb*F, *psb*H, *psb*I, *psb*J, *psb*K, *psb*L, *psb*M, *psb*T, *psb*Z
Large subunit of Rubisco	1	*rbc*L
Proteins of large ribosomal subunit	11	*rpl*14, *rpl*16, *rpl*2 (x2), *rpl*20, *rpl*22, *rpl*23 (x2), *rpl*32, *rpl*33, *rpl*36
Subunits of RNA polymerase	4	*rpo*A, *rpo*B, *rpo*C1, *rpo*C2
Proteins of small ribosomal subunit	14	*rps*11, *rps*12 (x2), *rps*14, *rps*15, *rps*16, *rps*18, *rps*19, *rps*2, *rps*3, *rps*4, *rps*7 (x2), *rps*8
Ribosomal RNAs	8	*rrn*16 (x2), *rrn*23 (x2), *rrn*4.5 (x2), *rrn*5 (x2)
Transfer RNAs	36	*trn*A*-*UGC (x2), *trn*C*-*GCA, *trn*D*-*GUC, *trn*E*-*UUC, *trn*F*-*GAA, *trn*G*-*GCC, *trn*H*-*GUG, *trn*I*-*CAU (x2), *trn*I*-*GAU (x2), *trn*K*-*UUU, *trn*L*-*CAA (x2), *trn*L*-*UAA, *trn*L*-*UAG, *trn*M*-*CAU, *trn*N, *trnN-*GUU, *trn*P*-*UGG, *trn*Q*-*UUG, *trn*R*-*ACG (x2), *trn*R*-UCU*, *trn*S*-*GCU, *trn*S*-*GGA, *trn*S*-*UGA, *trn*T*-*GGU, *trn*T*-*UGU, *trn*V*-*GAC (x2), *trn*V*-*UAC, *trn*W*-*CCA, *trn*Y*-*GUA, *trnf*M*-*CAU
Conserved hypothetical ORFs	3	*ycf*1, *ycf*2 (x2)

**Figure 3.**
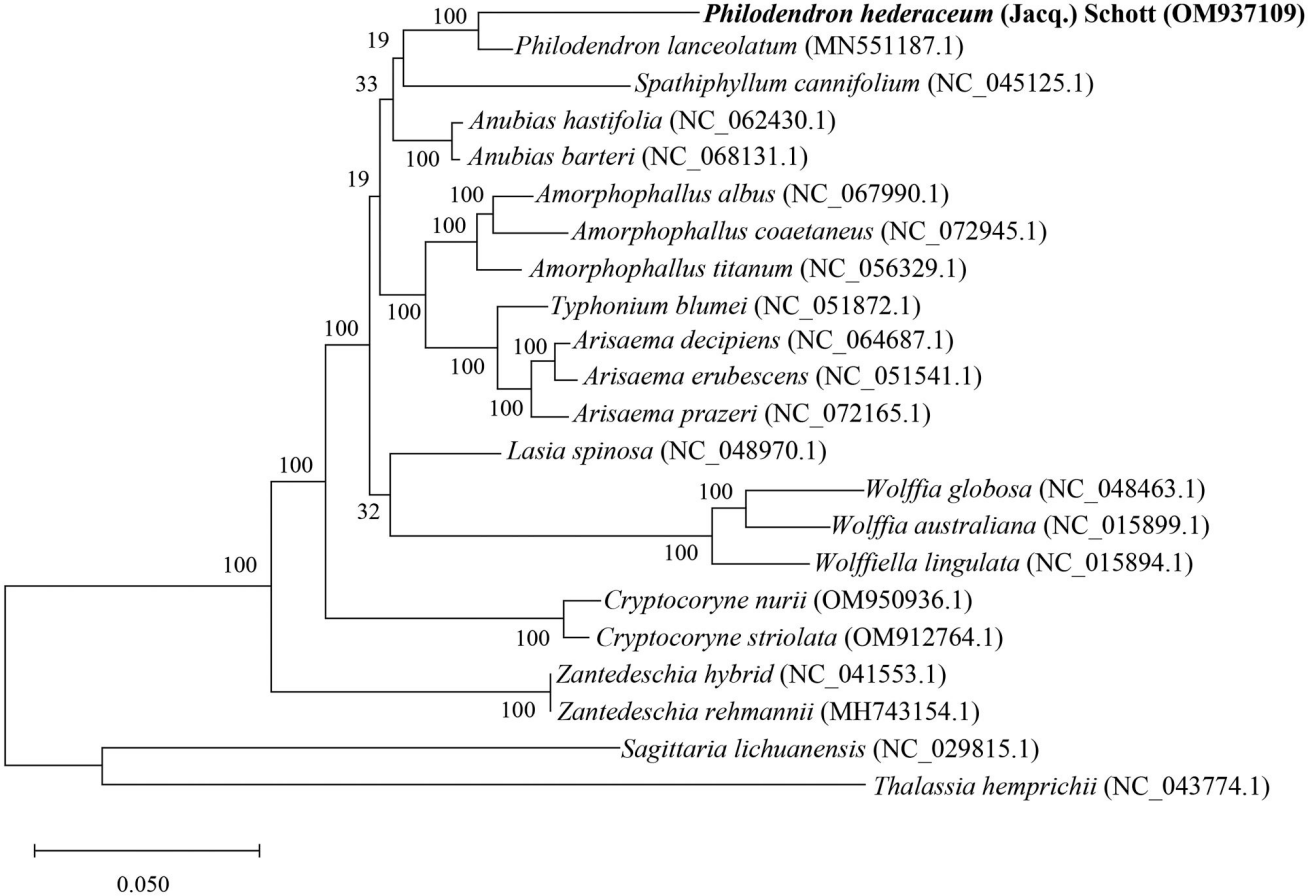
The maximum-likelihood phylogenetic tree of *Philodendron hederaceum* and its close relatives species based on complete chloroplast genome sequences. The complete chloroplast genome sequences of *Philodendron hederaceum* (bold font) and 19 related species from Araceae with two outgroup species, *Sagittaria lichuanensis* and *Thalassia hemprichii*, were downloaded from GenBank. These species include *Philodendron lanceolatum* (MN551187.1) (Henriquez et al. 2020), *Spathiphyllum cannifolium* (NC_045125.1) (Liu et al. 2019), *Anubias hastifolia* (NC_062430.1), *Anubias barteri* (NC_068131.1), *Amorphophallus albus* (NC_067990.1), *Amorphophallus coaetaneus* (NC_072945.1), *Amorphophallus titanum* (NC_056329.1), *Typhonium blumei* (NC_051872.1), *Arisaema decipiens* (NC_064687.1), *Arisaema erubescens* (Wall.) Schott (NC_051541.1) (Zhang et al. 2020), *Arisaema prazeri* (NC_072165.1), *Lasia spinosa* (NC_048970.1) (Abdullah et al. 2020), *Wolffia globosa* (NC_048463.1), *Wolffia australiana* (NC_015899.1) (Wang and Messing 2011), *Wolffiella lingulata* (NC_015894.1) (Wang and Messing 2011), *Cryptocoryne nurii* (OM950936.1), *Cryptocoryne striolata* (OM912764.1), *Zantedeschia hybrid* (NC_041553.1), *Zantedeschia rehmannii* (MH743154.1), *Sagittaria lichuanensis* (NC 029815.1) (Luo et al. 2016), and *Thalassia hemprichii* (NC_043774.1). The numerical value at each node represents bootstrap percentages based on 1000 replicates.

The phylogenetic analysis revealed that *P. hederaceum* is closely related to *Philodendron lanceolatum* within the family Araceae (Figure 3). The chloroplast genome of *P. lanceolatum* (MN551187) is 167,564 bp in length and contains 131 predicted genes (Henriquez et al. 2020), demonstrating a similar size in both length and number of genes. However, there are differences in several predicted genes between *P. hederaceum* and *P. lanceolatum*. Notably, for photosystem I and II assembly factor genes, three genes (*paf*I, *paf*II, *pbf*I) are present in *P. hederaceum*, while they are unidentified in *P. lanceolatum.* For subunits of photosystem II genes, *P. hederaceum* has 14 genes (*psb*A, *psb*B, *psb*C, *psb*D, *psb*E, *psb*F, *psb*H, *psb*I, *psb*J, *psb*K, *psb*L, *psb*M, *psb*T, *psb*Z), while *P. lanceolatum* has 15 genes. In terms of transfer RNA (tRNA) genes, *P. hederaceum* possesses 36 genes, whereas *P. lanceolatum* has 37 genes. Notably, *P. hederaceum* has single copy of *trn*N-GUU, whereas *P. lanceolatum* has two copies of *trn*N-GUU in the chloroplast genome. Regarding conserved hypothetical ORFs, *P. hederaceum* has three genes (*ycf*1, *ycf*2x2), whereas *P. hederaceum* has six genes (*ycf*1x2, *ycf*2x2, *ycf*3, *ycf*4).

## Discussion and conclusions

The complete chloroplast genome sequence of *Philodendron hederaceum* is 168,609 bp in size and contains 94,393 bp of LSC, 25,774 bp of SSC, and 24,221 bp of two IR regions. It harbors 85 protein-coding genes, 36 tRNAs, and eight ribosomal RNAs (rRNAs). Phylogenetic analysis involving *P. hederaceum* and 19 complete chloroplast genomes of the registered species in the Araceae family revealed *Philodendron lanceolatum* as its closest relative. Notably, there are differences in chloroplast gene content between *P. hederaceum* and its closely related species, *P. lanceolatum. P. lanceolatum* exhibits the absence of three photosystem-related genes, an additional copy of tRNA genes, and *ycf* ORFs compared to *P. lanceolatum* (Henriquez et al. 2020). This insight into the chloroplast genome of *P. hederaceum* is anticipated to facilitate further investigations into phylogenetic relationships and the evolution of chloroplast genes in *Philodendron*.

## Supplementary Material

Supplemental Material

Supplemental Material

## Data Availability

The data supporting this study’s finding are publicly available in GenBank at https://www.ncbi.nlm.nih.gov/genbank/, with the reference number OM937109. The BioProject, BioSample, and SRA numbers are PRJNA763941, SAMN21447885, SRR16016054 (Oxford Nanopore sequences), and SRR16016055 (Illumina sequences), respectively.
