# Supplementary figures and images for: The complete chloroplast genome of Philodendron hederaceum (Jacq.) Schott 1829 (Alismatales: Araceae)
Source: Mitochondrial DNA B Resour. 2024 Feb 9;9(2):262–6. doi: 10.1080/23802359.2024.2311748 (PMC10860476; doi:10.1080/23802359.2024.2311748)

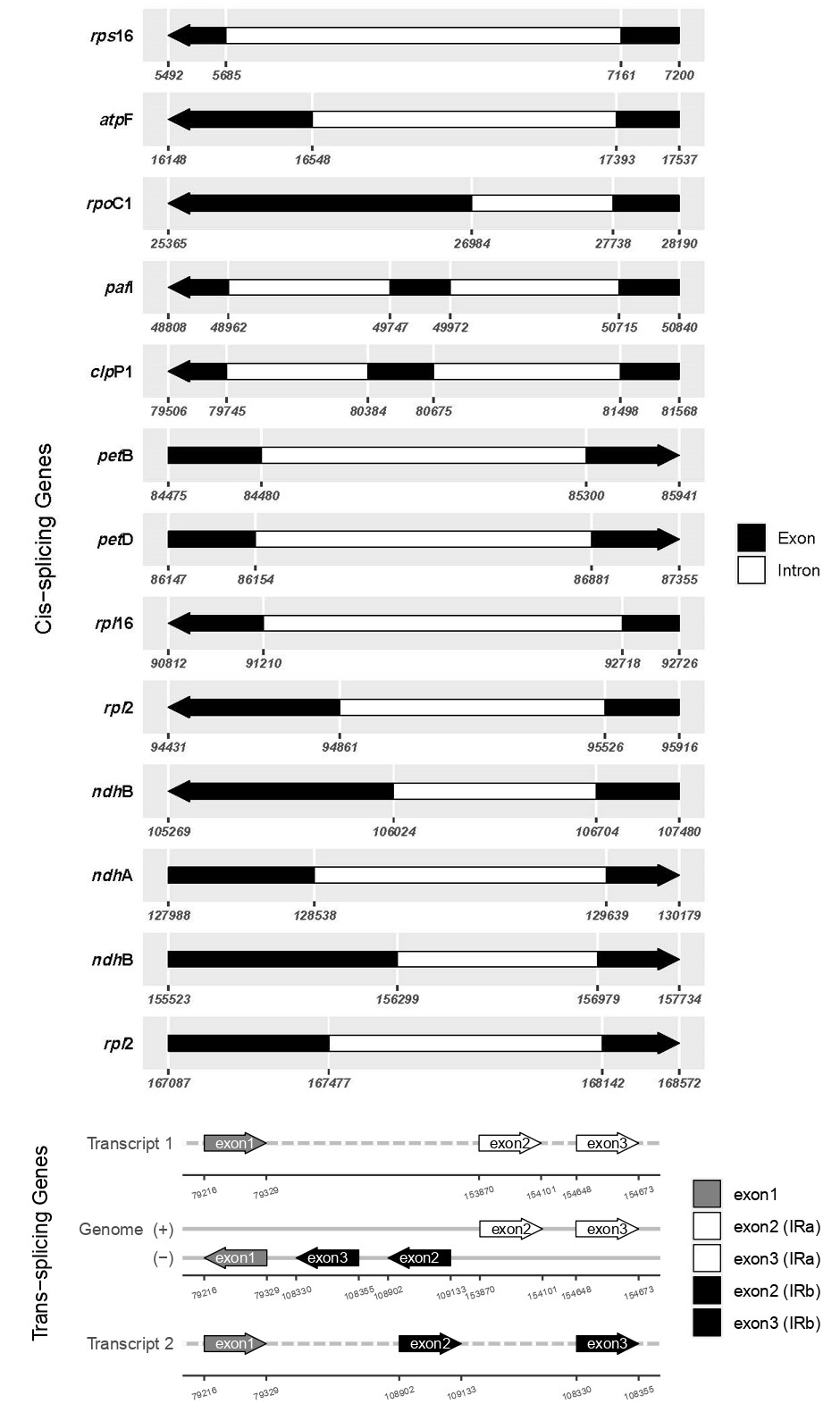

Supplement: Supplemental Material [file TMDN_A_2311748_SM3656.jpg]

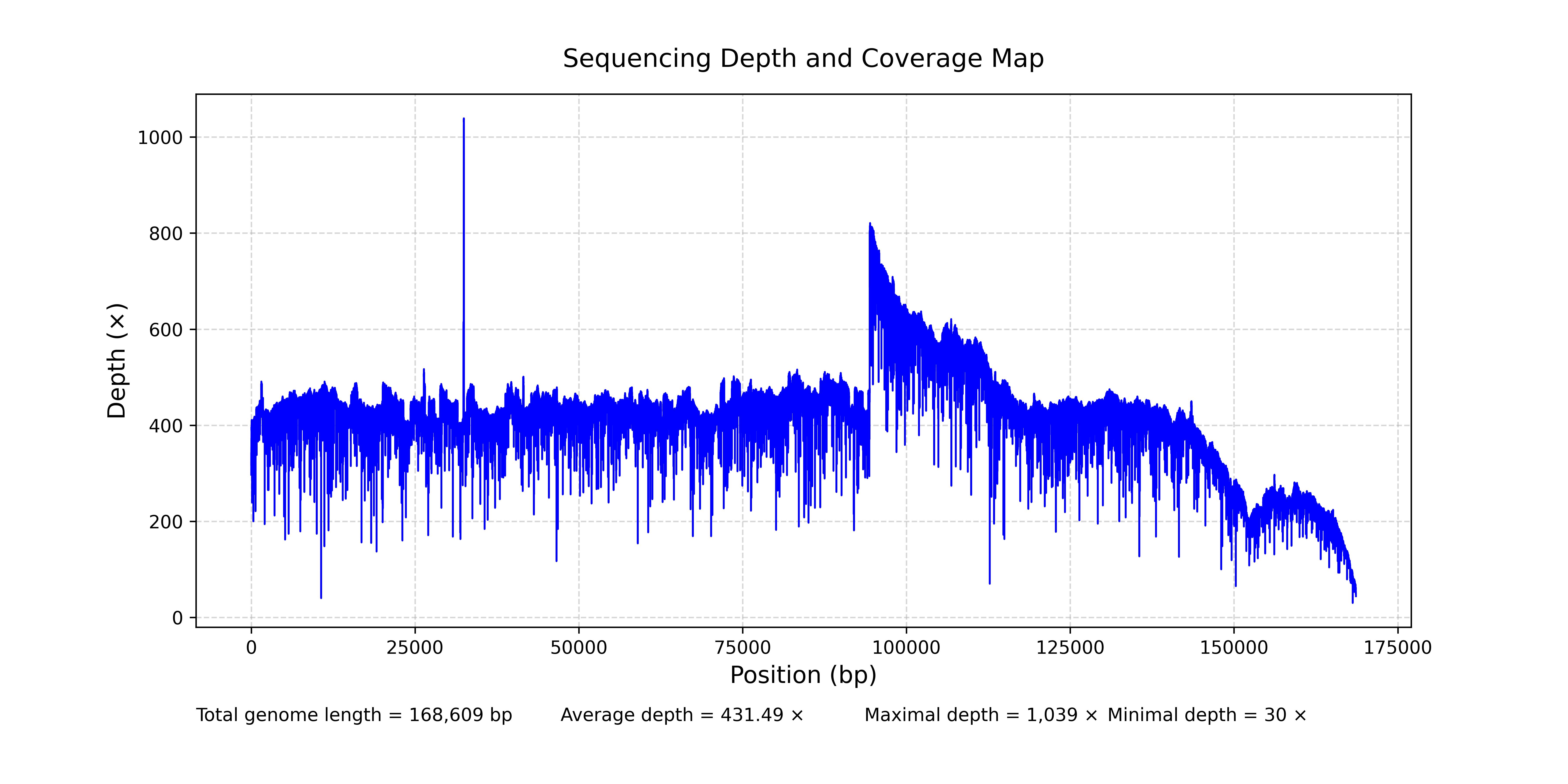

Supplement: Supplemental Material [file TMDN_A_2311748_SM3632.jpg]
